# Mathematical modeling of intratumoral immunotherapy yields strategies to improve the treatment outcomes

**DOI:** 10.1371/journal.pcbi.1011740

**Published:** 2023-12-19

**Authors:** Constantinos Harkos, Triantafyllos Stylianopoulos, Rakesh K. Jain

**Affiliations:** 1 Cancer Biophysics Laboratory, Department of Mechanical and Manufacturing Engineering, University of Cyprus, Nicosia, Cyprus; 2 Edwin L Steele Laboratories, Department of Radiation Oncology, Massachusetts General Hospital and Harvard Medical School, Boston, Massachusetts, United States of America; University of Southern California, UNITED STATES

## Abstract

Intratumoral injection of immunotherapy aims to maximize its activity within the tumor. However, cytokines are cleared via tumor vessels and escape from the tumor periphery into the host-tissue, reducing efficacy and causing toxicity. Thus, understanding the determinants of the tumor and immune response to intratumoral immunotherapy should lead to better treatment outcomes. In this study, we developed a mechanistic mathematical model to determine the efficacy of intratumorally-injected conjugated-cytokines, accounting for properties of the tumor microenvironment and the conjugated-cytokines. The model explicitly incorporates i) the tumor vascular density and permeability and the tumor hydraulic conductivity, ii) conjugated-cytokines size and binding affinity as well as their clearance via the blood vessels and the surrounding tissue, and iii) immune cells—cancer cells interactions. Model simulations show how the properties of the tumor and of the conjugated-cytokines determine treatment outcomes and how selection of proper parameters can optimize therapy. A high tumor tissue hydraulic permeability allows for the uniform distribution of the cytokines into the tumor, whereas uniform tumor perfusion is required for sufficient access and activation of immune cells. The permeability of the tumor vessels affects the blood clearance of the cytokines and optimal values depend on the size of the conjugates. A size >5 nm in radius was found to be optimal, whereas the binding of conjugates should be high enough to prevent clearance from the tumor into the surrounding tissue. In conclusion, development of strategies to improve vessel perfusion and tissue hydraulic conductivity by reprogramming the microenvironment along with optimal design of conjugated-cytokines can enhance intratumoral immunotherapy.

## Author summary

Cytokines are signaling proteins that can activate the immune response. Intratumoral administration of cytokines has shown promise in improving efficacy of cancer treatment. However, potential toxicity may still result from cytokines release from tumor into systemic circulation. Here, we developed a mechanistic mathematical model of cytokines transport in the tumor and the subsequent immune response. The model investigates the spatiotemporal distribution of intratumorally administered conjugated-cytokines in the tumor and the treatment outcomes, based on the physical and physiological characteristics of the conjugated-cytokines and the tumor microenvironment, respectively. Our results show the importance of tumor blood flow, vascular permeability and tumor tissue hydraulic conductivity in the treatment outcome. Moreover, we found that the size and binding affinity of the conjugated-cytokines are important determinants of the treatment outcome. Our model also shows how reprogramming the tumor microenvironment can improve the treatment outcome of intratumorally-injected cytokines.

## Introduction

Immune checkpoint inhibitors (ICIs) have transformed the treatment of cancer. To date 8 different ICIs have been approved alone or in combination with other therapies for ~80 indications [1]. However, less than 20% of patients currently benefit from these treatments [2]. Moreover, many patients develop immune-related adverse events, some of which can be fatal [3]. The abnormal and immunosuppressive tumor microenvironment (TME) not only hinders the delivery of ICIs, but also renders them ineffective once they accrue in tumors [4]. One approach to overcome these challenges is to normalize the tumor vasculature and microenvironment using anti-angiogenic agents [5]. Indeed 7 different combinations of ICIs and anti-angiogenic agents have been approved by the US FDA recently and multiple trials are currently testing this approach [6,7]. Another approach to improve the outcome of immunotherapies is the direct injection of immunostimulatory agents, tethered to a polymer or another substrate, into the tumor [8–10]. Agents being evaluated for this purpose include pro-inflammatory cytokines, such as, interleukin 2 (IL2) and interleukin 12 (IL12) [11–13].

**Fig 1:**
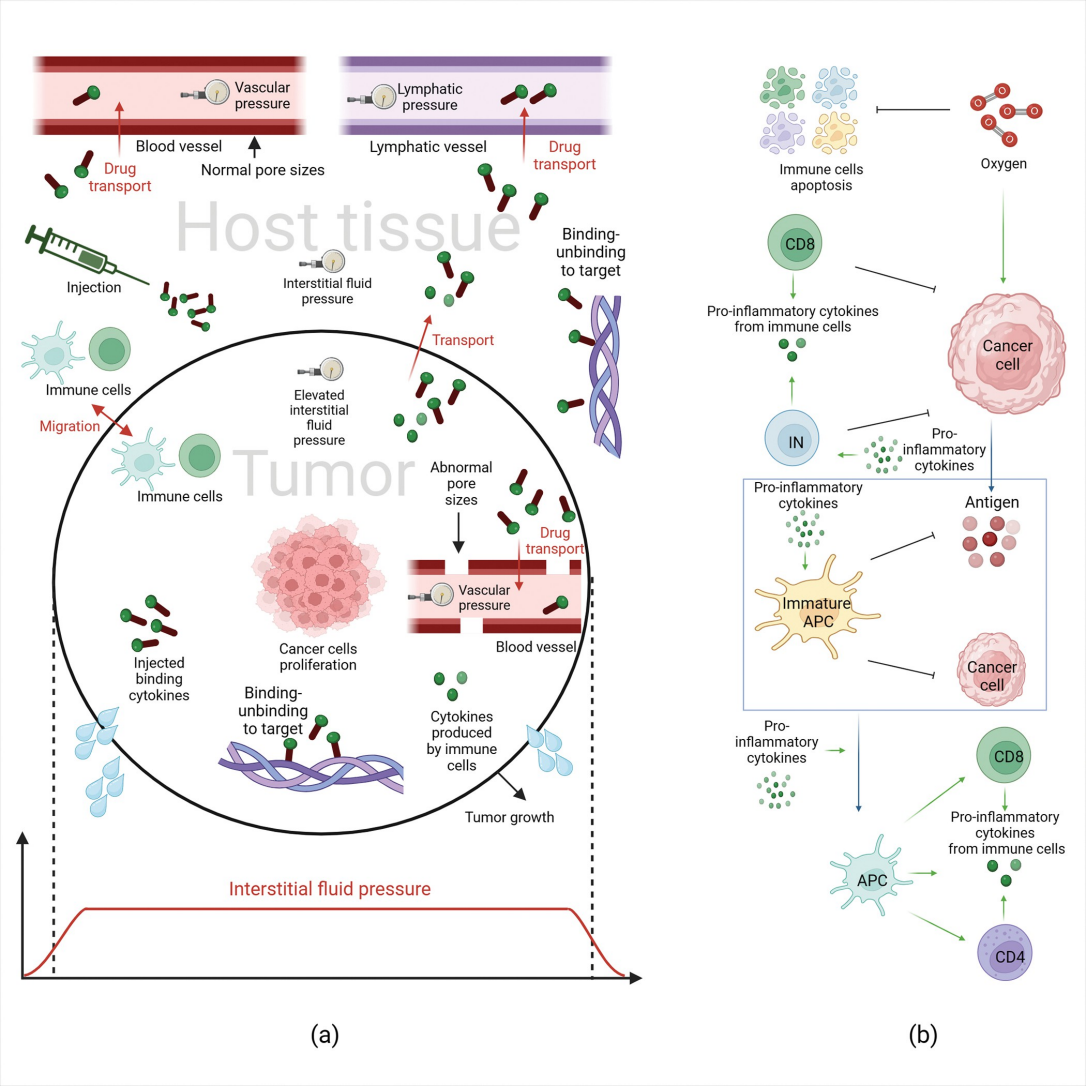
Model methodology. (a) Shcematic of various transport mechanisms considered in the model. The conjugated-cytokines are injected in the tumor region and can be transported via convection and diffusion to the host tissue and across the tumor vessel walls. Hyperpermeability of the tumor blood vessels and the lack of functional lymphatic vessels elevates interstitial fluid pressuse, inducing pressure gradients at the tumor periphery that drive transport of the conjugated-cytokines from the tumor to the host tissue via convection. The injected conjugated-cytokines can bind and unbind to the target (e.g., collagen fibers) in both tumor and host tissue. Cytokines produced by the immune cells can disperse via convection and difusion as well. Also immune cells can migrate (i.e., diffuse) from the tumor tissue to the host tissue and the reverse depending on the concentration gradients. (b) Model components of the immune system: IN represents the innate immune cells that induce cytolysis and produce antigen, e.g., Natural Killer cells. Immature APCs are the immature antigen presenting cells that can become antigen presenting cells (APCs). CD4 and CD8 represent effector CD4+ and CD8+ T cells. Production and activation of immune cells are affected by cytokines. The immune cells also produce cytokines. Oxygen supply increases cancer cells’ proliferation and tumor growth and decreases the apoptosis rate of the immune cells. Created with BioRender.com.

The goal of intratumoral injection of pro-inflammatory cytokines is to maximize their activity within the tumor while minimizing systemic exposure. After the cytokines are administered intratumorally, they can be cleared via the tumor-associated vasculature and the lymphatic system as well as escape from the tumor margin into the surrounding host tissue, resulting in potentially toxic levels in the circulation and the host organ [14–16]. A promising approach to increase tumor exposure and reduce adverse effects to normal tissues, is controlled release of cytokines from a polymer-conjugate injected into a tumor. One example of this approach is to fuse cytokines to collagen binding proteins, so that they are bound to collagen fibers within the tumor and do not clear rapidly from the tumor margin or by the vasculature. This strategy has been successful in enhancing treatment efficacy in preclinical studies [17–19]. Apart from the binding properties of the cytokine agent, its local exposure depends also on properties of the TME [20–24]. Specifically, the uniformly elevated interstitial fluid pressure (IFP) within the tumors (resulting from vascular hyperpermeability and dysfunctional lymphatics) decreases to normal values at the tumor-host tissue margin, causing steep pressure gradients at the tumor periphery and resulting in fluid flux from the tumor into the surrounding tissue. This can wash out the injected cytokines and reduce their concentration in the tumor region (Fig 1A) [20–24]. In addition, the hyper-permeability of tumor vessels might lead to the intravasation of the conjugated-cytokines into the vessels and their clearance via the circulation, a process that depends not only on the pore size of the vessel walls but also on the size, charge, configuration, and binding characteristics of the conjugated-cytokines [25–27]. Despite their importance in the effectiveness of intratumoral injection of cytokines, the role of these tumor parameters (i.e., vessel permeability, hydraulic conductivity, vessel density) and properties of the conjugated-cytokines (i.e., binding affinity, size) and their effects on the efficacy of intratumor injection of cytokines remain unexplored.

We have previously developed mathematical models of fluid and solute transport in tumors to investigate the role of vascular permeability, diffusion coefficient and hydraulic conductivity, binding and metabolism, interstitial fluid pressure, solid stresses as well as lymphatics [23,28–32]. Other *in silico* studies have examined the interactions of immune cells with cancer [33,34]. In addition, a recent intratumoral injection model examined the optimal cytokine design that increases intratumoral activity [18]. Although this model incorporated the binding-affinity of the conjugated-cytokines to their target, their transport into the blood circulation accounting for the conjugated-cytokines size and affinity, as well as temporal changes in model variables, they did not account for pathophysiological features and the spatial heterogeneity of the TME and the surrounding host tissue. To this end, building on our previous work, here we developed a mathematical model for intratumoral injection of conjugated-cytokines that accounts for i) spatiotemporal variations in model parameters, ii) the vascular and lymphatic function, iii) the hydraulic conductivity of the tumor and host tissue, iv) the interstitial fluid pressure, v) convection and diffusion within the tumor, from the tumor interstitial space to the blood vessels and the surrounding tissue, accounting explicitly for the size of the conjugated-cytokines, their binding affinity and vascular permeability, and vi) immune cells and cancer cells interactions (Table A in S1 Text). Two conjugated-cytokines cases were modeled: i) cytokines fused with mouse serum albumin (MSA) conjugated to the collagen binding protein, lumican [17], and ii) cytokines bound to aluminium hydroxide (alum) via ligand exchange between hydroxyls on the surface of alum and phosphoserine residues tagged to the cytokine by an alum-binding peptide [35]. We first assessed the robustness of our model by comparing model predictions with tumor growth data from these two independent studies [17,35]. Subsequently, we used the model to investigate the effect of the conjugated-cytokines size and binding affinity in conjunction with properties of the TME, on the efficacy of intratumorally injected conjugated-cytokines in reducing tumor growth. We further analyzed spatiotemporal changes in the concentration of the conjugated-cytokines and immune cells for a better understanding of the underlying mechanisms.

## Materials and methods

A brief description of the basic components of the mathematical framework is presented here. A detailed description of the equations that form the mathematical model is found in S1 Text.

The modeling framework consists of two steps. We first model the short time period immediately after the injection of the conjugated-cytokines from the needle into the tumor. Then, after the removal of the injection needle, we model the transport of the conjugated-cytokines into the tumor, its clearance through the blood vessels and tumor margin, as well as the growth of the tumor over a long time period. The first model simulates the injection of conjugated-cytokines inside a spherical tumor surrounded by host tissue (Fig 1 and Fig A in S1 Text). The conjugated-cytokine concentration profiles developed after the injection from the needle are used as initial conditions for the second model. The second model simulates cancer cell proliferation, the immune response and tumor growth (Fig 1 and Fig B in S1 Text). The model also accounts for transport of fluid and cytokines within the tumor, between the tumor and the host tissue as well as across the tumor vessel walls (Fig 1A). The model was developed and solved in COMSOL Multiphysics (COMSOL, Inc., Burlington, MA, USA) using the finite element method.

### Cytokine transport

The conjugated-cytokines can be in a free state or bound to the target (bound state). Both convection and diffusion are considered for the transport of the free conjugated-cytokines within the tumor and the host region. The diffusion coefficient of the conjugated-cytokines are determined by experimental data based on the conjugate size [36]. Also, the conjugated-cytokines that are not bound can intravasate into the vessels through diffusion and convection based on Starling’s approximation for mass transfer [28,30,37]. The transport properties of the conjugated-cytokines across the tumor vessel wall (i.e., vascular permeability and reflection coefficient) are determined explicitly by the relative ratio of the conjugate size to the size of the pores of the vessel wall, so that we can account for tumors with low, moderately, and highly permeable vessels as well as for conjugated-cytokines of varying size. For each conjugated-cytokine case, cytokines fused with mouse serum albumin conjugated to lumican and cytokines bound to aluminium hydroxide, the molecular weight were taken from the respective study [17,35] to determine their diffusion coefficient and transport properties across the tumor vessel walls. The rate of clearance from blood was also determined by the conjugate size based on previous work [38]. Furthermore, due to the different conjugate design and the different nature of target (collagen vs alum) for each conjugated-cytokine case, the respective binding affinity was used. In addition to cancer cells, the model includes innate and adaptive immune cells. These cells produce pro-inflammatory cytokines in addition to the injected cytokine, so that the total population of pro-inflammatory cytokines includes the cytokines produced by the immune system, the injected conjugated-cytokines that are free to move and the injected conjugated-cytokines that are bound. The total pro-inflammatory cytokines can enhance the immune system’s response to kill cancer cells and reduce tumor growth. The types of immune cells and immune cell–cancer cell interactions considered in our model are shown in Fig 1B and described below.

### Immune response

The simulation starts with a highly immunosuppressed TME by assuming initially no antigen presenting cells (APCs) or activated effector cells (e.g., effector CD4+ and CD8+ T cells), and predicts how the function of immune cells with positive effect on killing cancer cells impacts tumor growth. Due to the complex nature of the immune system and the immune cells—cancer cells interactions, we considered the immune cells in certain categories for simplicity. These include innate and adaptive immune cells. The innate cells are divided into two categories: cells that can induce cytolysis, such as Natural Killer (NK) cells, this category of cells can kill cancer cells and produce antigen, and the immature antigen presenting cells (IAPCs) that includes the dendritic cells and a sub-set of macrophages. When IAPCs interact with cancer cells or antigens they become APCs. The higher the number of APCs the more CD4+ and CD8+ T cells will reach the tumor and host tissue. In addition, and for simplicity, we did not include an explicit model of lymph nodes for the activation of T cells. Instead, we assumed that T cell activation takes place external to the tumor in lymph nodes where the T cells encounter APCs, but the activated T cells return to the same location in the tumor from which the APCs depart. Effector CD8+ T cells kill cancer cells and further increase the concentration of antigens in the region. Both CD4+ and CD8+ T cells produce pro-inflammatory cytokines to further increase the immune response (Fig 1B).

### Interstitial fluid flow

Fluid flow within the tumor and host tissue is governed by Darcy’s law, taking into account the displacement of both the tumor and the surrounding normal tissue due to the growth of the tumor. Continuity of fluid velocity and fluid flux is applied at the tumor/host tissue interface [30]. The model also accounts for fluid flux across the tumor vessel walls based on Starling’s approximation [22,30,39,40]. The hydraulic conductivity of the tumor vessel wall is calculated based on the vessel walls pore size, following our previous research [28,39].

### Oxygen transport

The model considers oxygen transport from the vessels into the tumor and host tissue and transport within the tissue. Overall tumor growth depends on cancer cell number (concentration), which is determined by cancer cell proliferation (as a function of tissue oxygenation) and cancer cell killing by immune cells [41,42]. Details about the model variables as well as the baseline and initial values of the model parameters are given in Tables B and C in S1 Text.

## Results

### Model validation and determination of model parameters values

**Fig 2:**
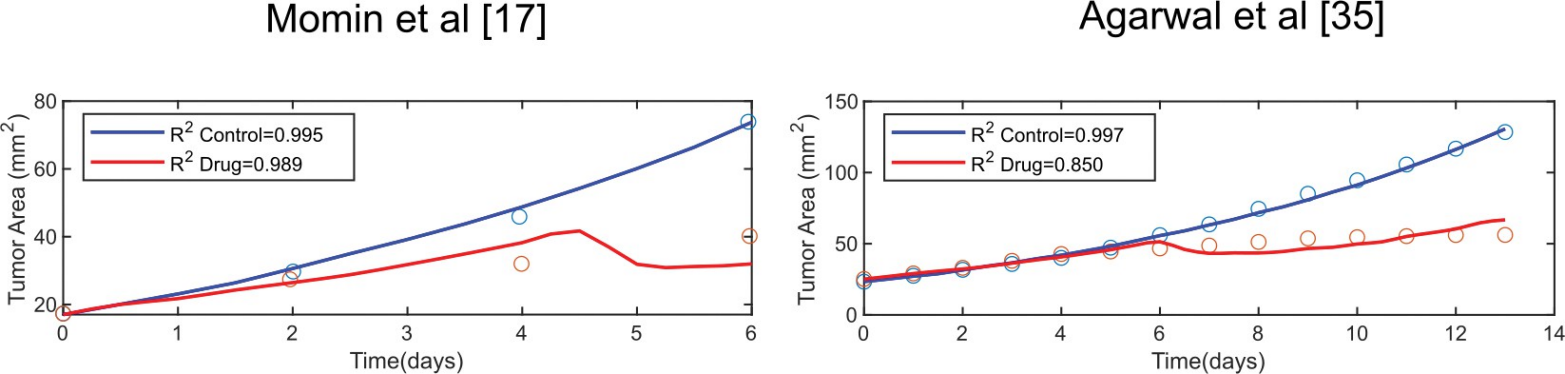
Experimental data (circles) of tumor growth and model predictions (solid line) for control tumors (blue) and tumors treated with intratumoral injection of conjugated-cytokines (red) by Momin et al. [17] and Agarwal et al. [35].

The values of the model parameters that could not be obtained from previous studies (Table C in S1 Text) were determined by fitting the model to tumor growth data from two published studies [17,35]. These studies included a control group that did not receive any treatment (control) and a group that received intratumoral injection of conjugated-cytokines as a drug. For the control groups, the variables related to the injected conjugated-cytokines become zero so that the pro-inflammatory cytokines in the tissue are produced only by the immune cells (Eq (30) in S1 Text). We did not consider any other variation of model parameters between the control and injected cytokines group. All tumor growth curves were fitted simultaneously to optimize the global fit. An optimization algorithm in MATLAB (The Mathworks, Inc., Natick, MA, United States) using the COMSOL with MATLAB interface was employed for the fitting. More information about the optimization and the optimized parameters can be found in the S1 Text. As shown in Fig 2 the model can reproduce tumor growth data with a good accuracy (R^2^ ~ 1).

**Fig 3:**
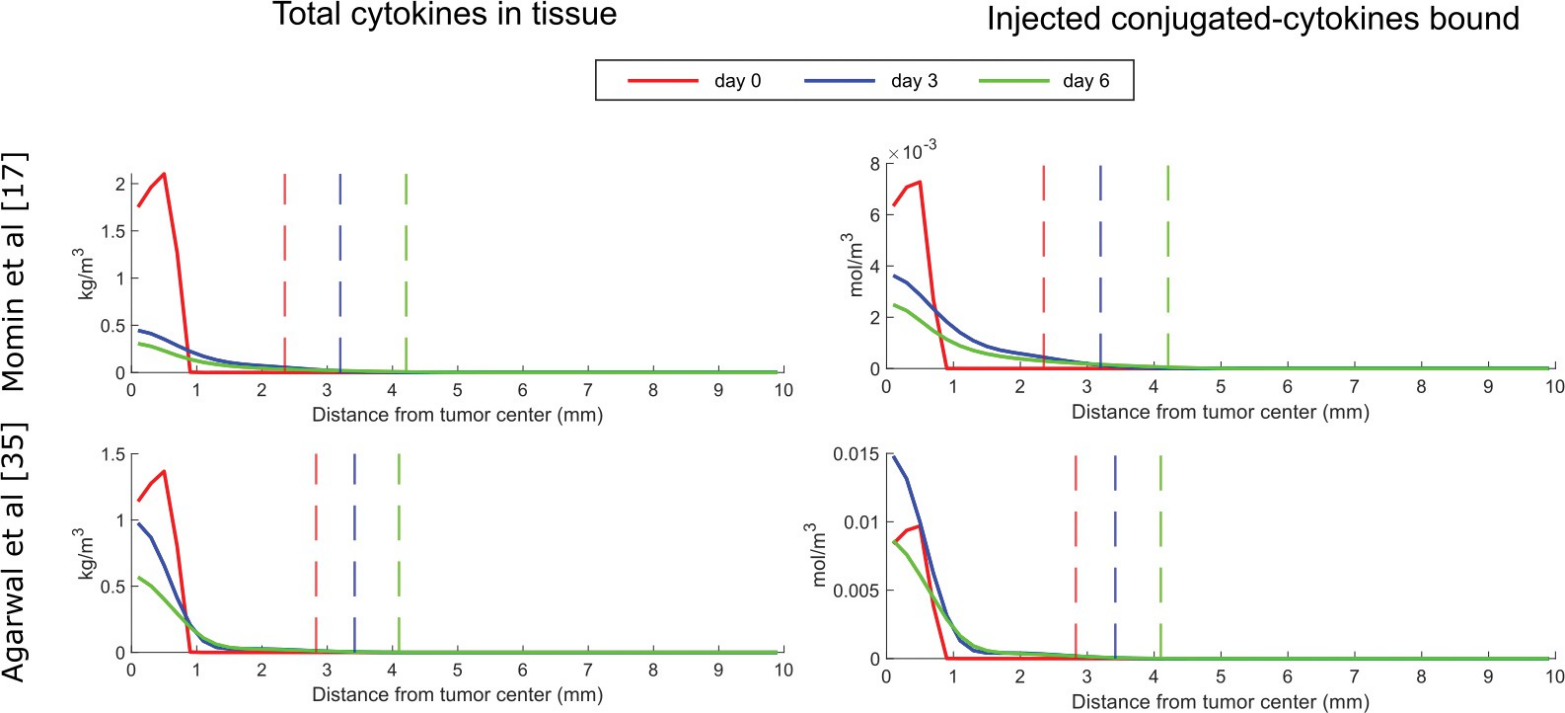
Results for the total amount of cytokines and the bound conjugated-cytokines for various time points for each study. The plots represent the distribution in the radial direction. The value 0 in the x axis corresponds to the tumor center. As we move along the x axis, we move away from the tumor center towards the host tissue. Plots include both the tumor region and host tissue that surrounds the tumor. The vertical dashed lines show the tumor boundary at the given time points.

**Fig 4:**
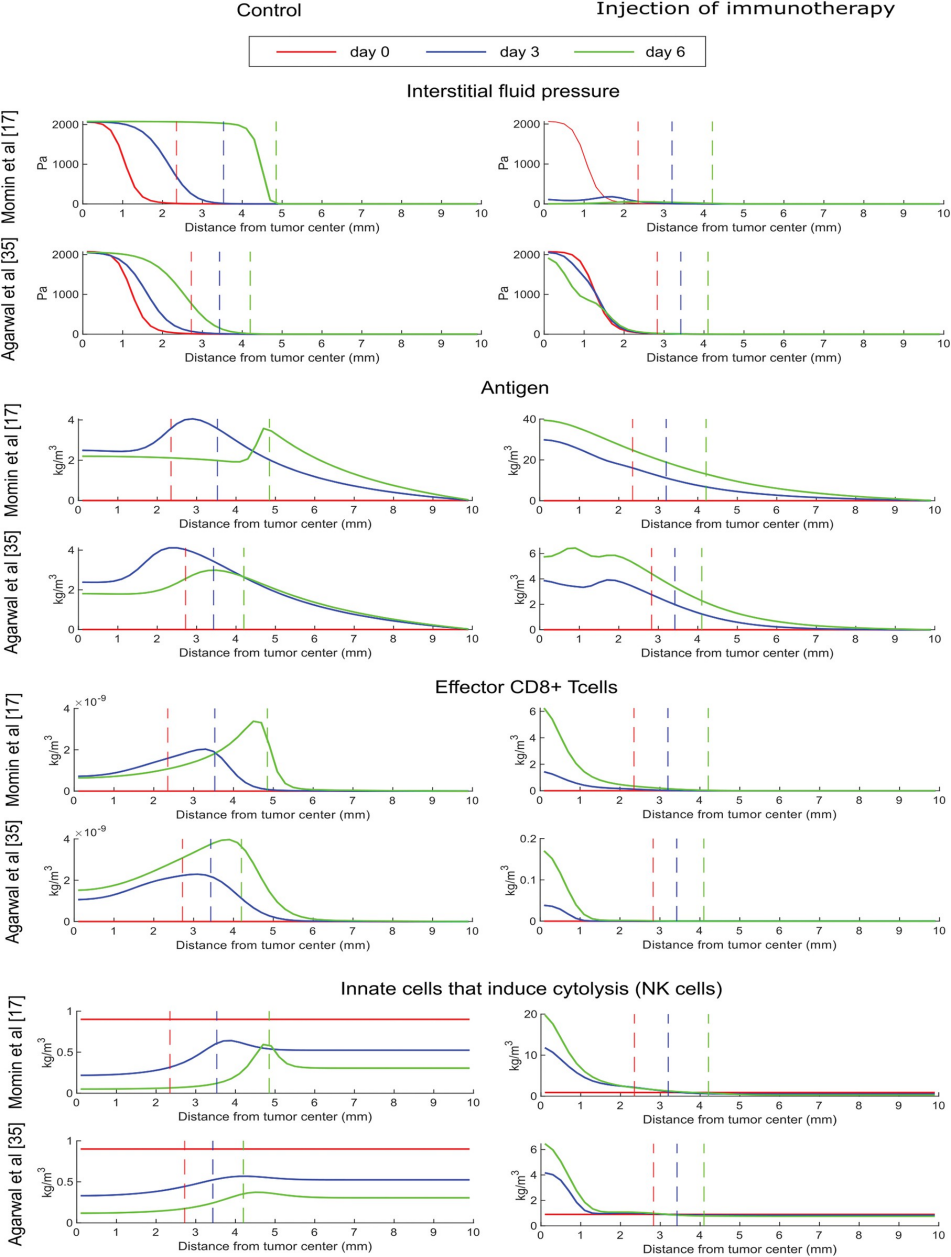
Results at various time points for each optimization case. The plots represent the distribution in the radial direction. The value 0 in the x axis corresponds to the tumor center. As we move along the x axis, we move away from the tumor center towards the host tissue. Plots include both the tumor region and host tissue that surrounds the tumor. The vertical dashed lines represent the tumor boundary.

Due to the complexity of the model that includes various interactions and mechanisms, the behavior of the model variables is not intuitive. Thus, we generated plots to further investigate the changes in the model variables that led to the reduction of the tumor growth after the injection of therapy. Model predictions for the spatial distribution of cytokines are presented in Fig 3, whereas predictions for IFP, antigen concentration, CD8+ T cells and NK cells are presented in Fig 4 for both studies. Day 0 corresponds to the time of the intratumoral injection of the conjugated-cytokines. The concentration of the total cytokines decreased after the injection as expected.

The IFP was elevated within the tumor, reaching the levels of microvascular fluid pressure at the tumor center and droped to normal values at the tumor margin (Fig 4, control). This spatial distribution of IFP created a fluid flux at the tumor margin towards the host tissue, resulting in increased concentration of antigen, effector CD8+ T cells and NK cells at the interface of the tumor with the host tissue compared to the tumor interior (control group). Intratumoral injection of cytokines can reduce the IFP levels, which is more evident in the case of Momin et al.[17] where the efficacy of the treatment is more pronounced and induced considerably higher amounts of innate and adaptive immune cells compared to the respective control cases. In the treatment case, the spatial distribution of immune cells changed compared to the control and most immune cells can be found at the center of the tumor where the concentration of cytokines and antigens is the highest.

### Dependence of treatment efficacy on conjugated-cytokines properties

**Fig 5:**
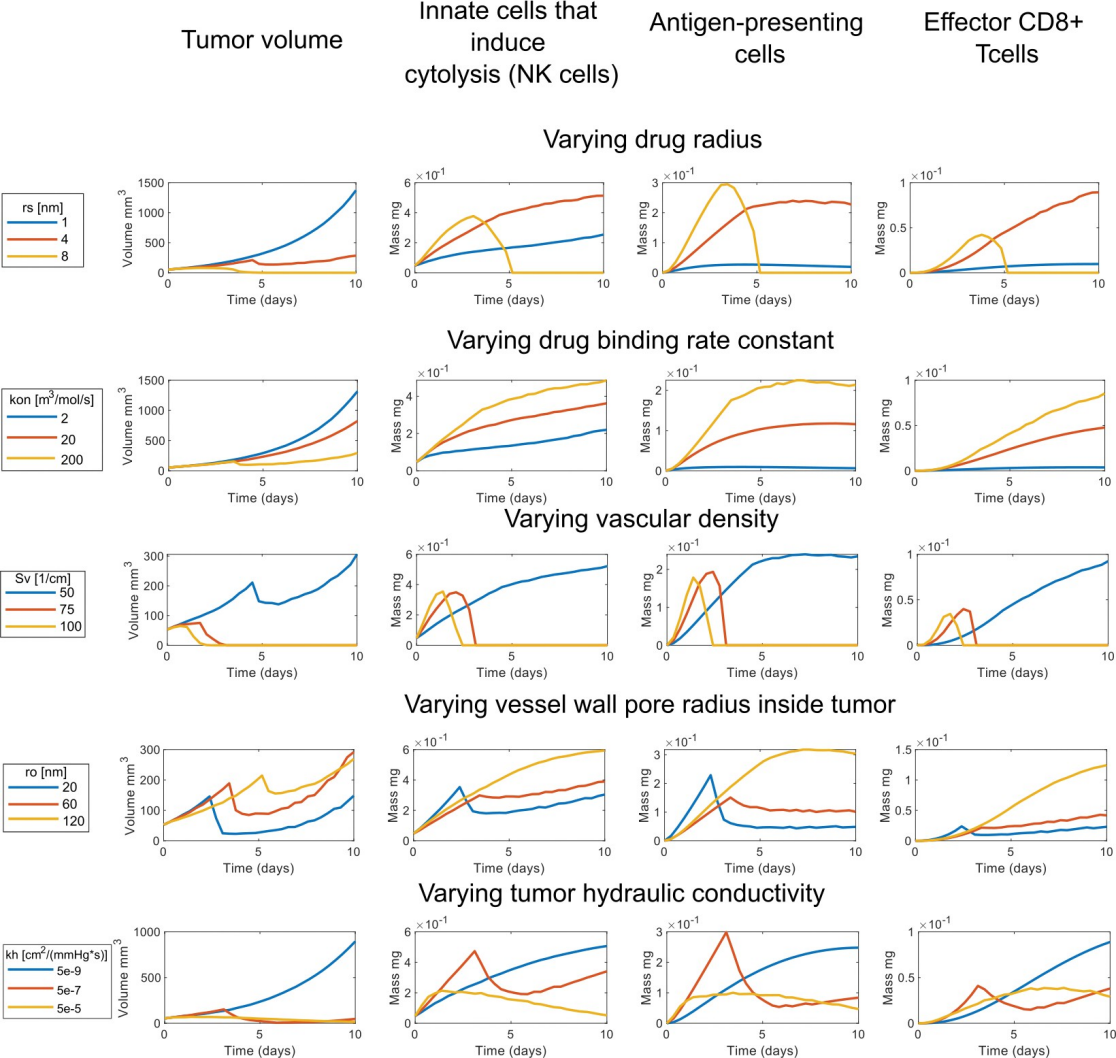
The impact of various model components to tumor growth by varying a single parameter. Figure presents the tumor growth through time and the number of innate cells that induce cytolysis (NK cells), antigen presenting cells and effector CD8+ T cells when varying: the injected conjugate radius, the conjugate binding rate constant, the vascular density inside the tumor region, the vessel wall pore radius inside tumor, and the hydraulic conductivity inside the tumor region. The baseline values of the parameters for these simulations are: rs = 3.85[nm], kon = 100 [m^3^/mol/s], Sv = 50[1/cm], r_0_ = 100 [nm], kh = 4.13e-8 [cm^2^/mmHg/s].

Subsequently, we aimed to investigate how changing the properties of the conjugated-cytokines can affect the efficacy of treatment. Specifically, we varied the size and binding affinity of the drug and the model predictions are presented in Fig 5 for varying the conjugated-cytokines radius, rs, from 1 to 8 nm and when the binding rate constant, kon, is increased/decreased by an order of magnitude.

Changes in both the size of conjugated-cytokines from 1 to 8 nm in radius and the binding rate constant from 2 to 200 m^3^/mol/s altered the tumor growth rate and induced significant changes in the number of immune cells. Cytokine conjugates of small size were cleared fast from the tumor owing to increased diffusion within the tumor and intravasation into blood vessels and thus, cannot induce a significant anti-tumor immune response. Increasing the size of the drug to 4–8 nm in radius dramatically reduced tumor volume and even eliminated tumor. Increases in binding rate constant hindered the clearance of the cytokines and thus, improved anti-tumor immune responses, by increasing the number of intratumoral CD8+ T cells soon after intratumoral administration of cytokines.

### Role of the tumor microenvironment in treatment efficacy

Next, we set out to study how varying the physical and physiological properties of the TME can improve the efficacy of injected conjugated-cytokines. Specifically, we varied the vascular density and tumor vessel wall permeability (i.e., the size of the pores in the tumor vessel walls) as well as the hydraulic conductivity of the tumor. The tumor functional vascular density was varied from 50 to 100 cm^-1^ [43], the radius of the pores of the tumor vessel walls from 20 nm to 120 nm [44,45], and the tumor hydraulic conductivity from 5x10^-9^ to 5x10^-5^ cm^2^/mmHg-s [45]. As shown in Fig 5, a 50% increase in the functional vascular density and thus, tumor perfusion, was sufficient to potentiate anti-tumor immunity. In the model and in agreement with the literature, increase in perfusion increased the number of immune cells in the tumor at early times after cytokines injection (Fig 5), which led to complete tumor elimination. Subsequently the immune cells left the tumor and their numbers go down to zero. Elimination of tumor is also predicted when the hydraulic conductivity of the tumor was increased. The increase in the tumor hydraulic conductivity increased the interstitial velocity and thus, allowed for better penetration of the conjugated cytokines in all regions of the tumor. This resulted in a robust anti-tumor immune response and a dramatic reduction in tumor volume.

Finally, the vessel wall pore size determined the transport of the conjugates across the tumor vessel wall. Tumors hinder the transport of nano-sized drugs across the tumor vessels [27]. Model predictions agree with previous findings in that tumors with more permeable vessels allowed the transvascular transport of nano-sized therapeutics and in our case allowed the clearance of the conjugated cytokines, which reduced treatment efficacy (Fig 5). Interestingly, the model predicted that even though the tumor responded to therapy at early times after cytokines administration and thus, the tumor volume decreased, at longer times the tumor regrew, which implies the need for repeated intratumoral administration of cytokines. Interestingly, vascular normalization strategies aim to reduce vessel permeability to large molecule/nanoparticles, whereas stroma normalization strategies improve tumor hydraulic conductivity, in both cases improving perfusion [46].

**Fig 6:**
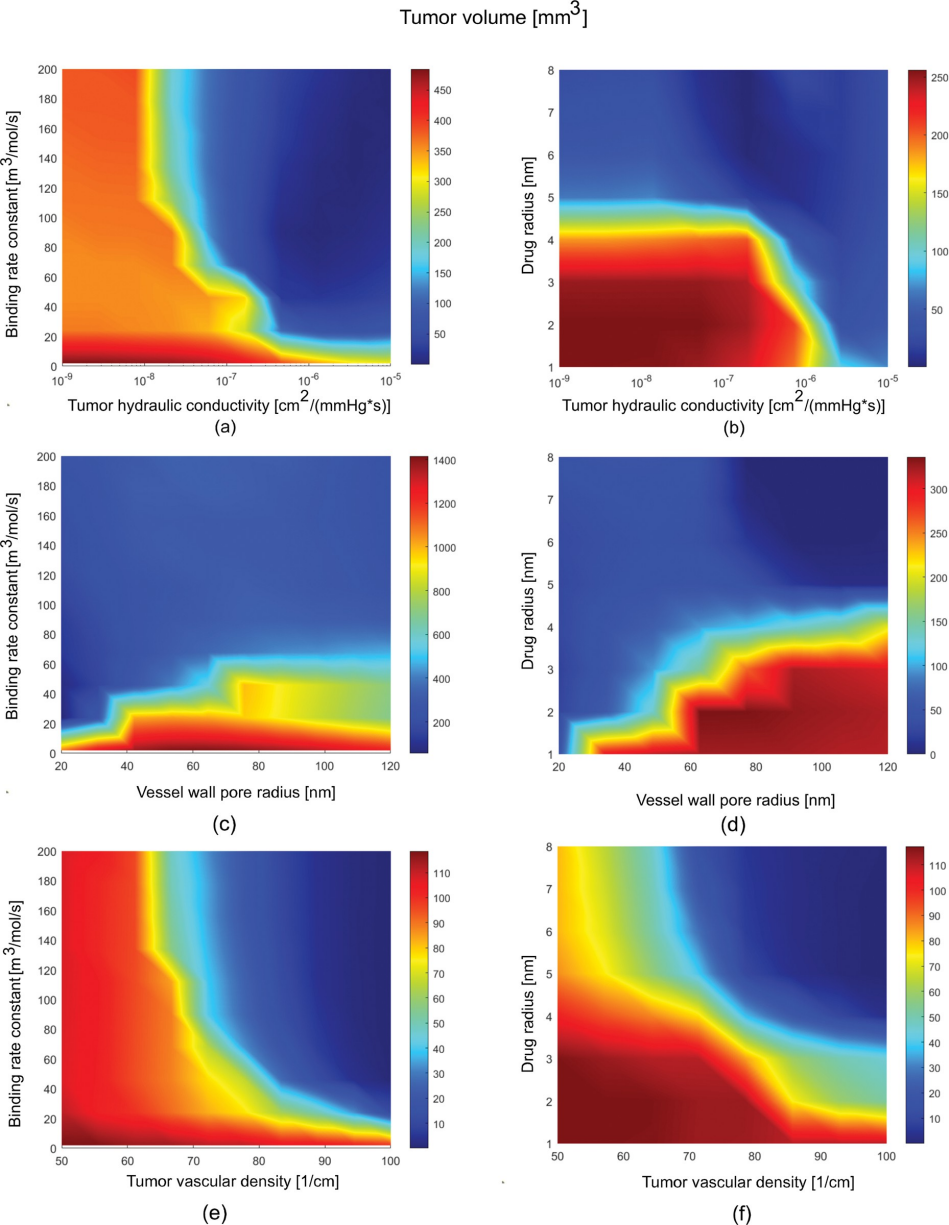
Diagrams of the efficacy of conjugated-cytokines injection as a function of tumor physiological properties and conjugate radius and binding affinity. Each point in the diagrams represents the tumor volume of a different simulation. The tumor volume is measured either at the end of the simulations (day 10) or at the time point where at least one of the simulations reached complete cure (i.e., tumor volume becomes zero). For each simulation only the parameters shown in the two axes were varied. (a) The hydraulic conductivity in the tumor region was varied relative to the binding of the injected conjugate (day 7.5) and (b) the conjugate radius (day 5.2). (c) The tumor vessel wall pore radius was varied relative to the binding of the injected conjugate (day 10) and (d) the conjugate radius (day 6.0). (e) The tumor vascular density was varied relative to the binding of the injected conjugate (day 3.2) and (f) the conjugate radius (day 2.9).

To further investigate the effect of the properties of the TME and the injected conjugated-cytokines, we varied two parameters simultaneously to generate tumor volume diagrams as shown in Fig 6. From these diagrams, firstly, we conclude that increasing the tumor hydraulic conductivity enhanced the efficacy of conjugated cytokines even of small size and low binding affinity (Fig 6A and 6B). Furthermore, increasing the size of the drug and thus, decreasing both the diffusion of the conjugated-cytokines within the tumor tissue and their extravasation into the blood vessels results in reduced tumor volumes for various values of the hydraulic conductivity. Interestingly, increasing the drug size for a tumor with low hydraulic conductivity can induce a similar effect with a smaller drug in a tumor environment with high hydraulic conductivity (Fig 6B). Additionally, reduced tumor volumes can be achieved for lower binding capabilities of the conjugated-cytokines by decreasing the vessel wall pores. Also, increasing the binding rate constant to more than 50 m^3^/mol/s can reduce tumor volume independent of the vessel wall pore size (Fig 6C). By also increasing the drug size we can achieve improved therapeutic efficacy independently from the vessel wall pore size as well (Fig 6D). Finally, increasing vascular density, while also increasing either the binding affinity or the size of the conjugated cytokines can enhance the efficacy of the treatment (Fig 6E and 6F). From all the analysis, can be inferred that conjugated-cytokines larger than 5 nm in radius with binding rate constant above 50 m^3^/mol/s can induce better therapeutic outcomes.

## Discussion

Our model simulations support the hypothesis that intratumoral injection of tethered cytokines is a promising strategy to control tumor growth. Previous mathematical models showed that by increasing molecular size and/or matrix-targeting affinity of the injected cytokines improves therapeutic efficacy [18]. Our study agrees with these findings, predicting that by increasing the molecular size, the effective diffusion of the injected conjugated-cytokines decreases and thus, they remain within the tumor at higher concentrations. Also, the exposure within the tumor region increases when increasing the binding affinity and thus, making it more difficult for the cytokines to escape from the tumor. Therefore, both molecular weight and binding will lower the effective diffusion rate of the injected drug and only convection can distribute the drug uniformly from the injection site to throughout the tumor. Additionally, our study extends the modeling framework by adding spatiotemporal variations in model parameters, pathophysiological properties of the TME, IFP gradients, convection-diffusion within the tumor and host tissue and across the vessel walls, and cancer-immune cells interactions. Our results suggest that these additional considerations shed further light on the outcome of the treatment. For example, incorporation of the immune system revealed that the injected conjugated-cytokines boost the activation of the adaptive immune cells and also support innate immune cells to further activate the adaptive immune system.

Our results also highlight the fact that normalizing pathophysiological features of the TME can improve therapeutic effects. Abnormal blood vessels is a hallmark of solid tumors [47]. Blood vessel abnormalities include hyperpermeability of the tumor vessel wall, as a result of increased levels of proangiogenic factors released under tumor hypoxic conditions, and/or vessel compression/collapse due to the accumulation of mechanical forces in the tumor [23,48]. In both cases, tumor vessel perfusion is reduced. Tumor hydraulic conductivity is often low in fibrotic, desmoplastic tumors, such as triple-negative breast cancer, pancreatic cancer and sarcomas. The excessive collagen matrix and hyaluronan in these tumors hinder the transport of fluid within the tumor interstitial space and thus, decrease the hydraulic conductivity. Stroma normalization strategies aim to target these components of the extracellular matrix either directly or by reprogramming cancer-associated fibroblasts. Therefore, stroma normalization can decompress vessels, improving functional vascular density and increasing the hydraulic conductivity of the tumor [23,48]. Increase in the hydraulic conductivity also enhances convective transport and makes the distribution of the conjugated-cytokines in the tumor more uniform. Our model simulations show that modulation of the TME to reduce vascular permeability, improves perfusion and increases hydraulic conductivity. These strategies should be considered to improve therapeutic outcomes of intratumorally injected cytokines. The strategy to normalize the TME should be tailored to its specific pathophysiological characteristics: abundant hyperpermeable vessels or abundant extracellular matrix or both. Our model simulations also agree with published data, highlighting that the conjugate size and binding capability have a large impact on the outcome of therapy. This is promising because by designing the optimal conjugate, the treatment could be improved. Furthermore, combination with a TME-normalizing strategy would further add to the efficacy of the treatment.

Although the model predicted reduced tumor growth due to the administration of conjugated-cytokines, at longer times the tumor recovered. Repeating intratumoral administration might further maintain therapeutic effects and increase efficacy. However, multiple injections might increase systemic accumulation of the conjugated-cytokines, leading to toxic effects [49]. Modulation of the TME and designing conjugated-cytokines with increased molecular size and/or matrix-targeting affinity reduces toxic accumulation and might increase the number of the permiting injections without causing toxic effects. In general, there might be a minimum time of exposure of a certain concentration of the conjugated-cytokines inside the tumor, for the therapy to be effective. This threshold could be akin to the Allee effect [50–52], where below a certain exposure time of this minimum concentration, the treatment is not effective enough to trigger a sufficient immune response to combat the cancer cells. There might be also a minimum exposure time of a certain concentration of the cytokines in the blood that causes toxicity. Thus, when considering intratumoral injection of conjugated-cytokines this level should not be exceeded. Both these thresholds may vary from patient to patient, which makes the development of a personalized adaptive therapy framework that includes the monitoring of the individual’s tumor and immune response a promising approach to optimize therapeutic effects.

Our model also has some limitations as we made several assumptions to keep the model simple. The tumor was assumed to grow as a sphere, which is not usually the case. In addition, the model did not account for the drug-conjugate surface charge and configuration, which along with the conjugate size, can affect its transport properties [48,53,54]. Furthermore, the vessel wall pore radius was assumed uniform, while there must be a distribution. Transport properties, such as the interstitial diffusivity of the conjugates, depend not only on their size but also on the density (i.e., porosity) of the tumor interstitial space that varies among tumor types [36]. In this study we did not consider changes in the diffusion coefficient of the conjugates due to variations among tumor types. We also assumed very few intratumor immune cells and none of them activated at the beginning of the simulation. This may not be the case for many tumors. Also, our model did not account for the fact that immune cells can secrete immunosuppressive cytokines. Furthermore, our model does not explicitly incorporate the draining lymph node and effector T cell priming or the cancer cells leaving the tumor via the blood vessels and peri-tumoral lymphatics. In principle, we can relax these assumptions by incorporating additional parameters into our model. However, this is likely to change the results only quantitatively, whereas the conclusions reached in this study related to the parameters that affect the efficacy of intratumoral injection of conjugated-cytokines would remain unchanged.

## Supporting information

S1 Text Detailed description of the equations that form the mathematical model. **It contains the following Figures and Tables. Fig A Computational domain with axial symmetry.** The domain includes the tumor region and the host tissue. The needle reaches the center of a spherical tumor. **Fig B Computational domain with spherical symmetry.** The domain includes the tumor region and the host tissue. The tumor grows as a sphere and deforms the host tissue. **Table A: Mathematical model characteristics compared to other models. Table B: Table of model variables. Table C: Table of model parameters.**
(PDF)
