# Supplementary material for: Mathematical modeling of intratumoral immunotherapy yields strategies to improve the treatment outcomes
Source: PLoS Comput Biol. 2023 Dec 19;19(12):e1011740. doi: 10.1371/journal.pcbi.1011740 (PMC10763956; doi:10.1371/journal.pcbi.1011740)
Supplement: S1 Text — It contains the following Figures and Tables. Fig A Computational domain with axial symmetry. The domain includes the tumor region and the host tissue. The needle reaches the center of a spherical tumor. Fig B Computational domain with spherical symmetry. The domain includes the tumor region and the host tissue. The tumor grows as a sphere and deforms the host tissue. Table A: Mathematical model characteristics compared to other models. Table B: Table of model variables. Table C: Table of model parameters. (PDF) [file pcbi.1011740.s001.pdf]

S1 Text: Mathematical modeling of intratumoral immunotherapy yields strategies to improve the treatment outcomes

Constantinos Harkos, Triantafyllos Stylianopoulos, Rakesh K. Jain

Modeling of intratumoral injection

Implementation of model equations

The model equations are solved using COMSOL Multiphysics (COMSOL, Inc. Burlington, MA, USA). At the tumor center, the initial cancer cell density was assumed to have its peak value and then it decreases alongside the straight line due to a step function. The domain above a threshold cancer cell density is assumed as the tumor region and below that, the host tissue.
The parameters change from their abnormal-tumor value to their normal value as a function of the cancer cell density. The values change at the threshold cancer cell density using a step function. The step functions are used to certify continuity of the model variables.
A 2D geometry with axial symmetry was modeled. The geometry includes the tumor, the host tissue and a 28 gauge needle reaching the tumor center. Conjugated-cytokines are injected from the needle outlet. Flux source was assumed for the needle outlet and no flux for the needle periphery.

Interstitial pressure-fluid velocity

Normal and tumor tissues have properties similar to those of a porous medium. Brinkman's equation describes the flow in porous medium in cases where the velocity gradients are non-negligible and reads:

$$\frac{\rho}{\epsilon_p}\frac{\partial \mathbf{v^f}}{\partial t} = -\boldsymbol{\nabla} p_i + \boldsymbol{\nabla}\cdot\left[\frac{\mu}{\epsilon_p}\Big(\boldsymbol{\nabla}\mathbf{v^f} + (\boldsymbol{\nabla}\mathbf{v^f})^{\tau}\Big) - \frac{2}{3}\frac{\mu}{\epsilon_p}(\boldsymbol{\nabla}\cdot\mathbf{v^f})\mathbf{I}\right] - \Big(\frac{1}{k_{th}} + \frac{Q_m}{\epsilon_p^2}\Big)\mathbf{v^f} \quad (1)$$

Where μ is the dynamic viscosity, ρ is the density, p_i is the interstitial pressure, $\mathbf{v^f}$ is the fluid velocity and k_{th} is the hydraulic conductivity of the interstitial space [24]. For incompressible fluid flow the conservation of mass reads:

$$\rho\boldsymbol{\nabla}\cdot\mathbf{v^f} = Q_m \quad (2)$$

$$Q_m = \rho\Big(L_p S_v(p_v - p_i) - L_{pl} S_{vl}(p_i - p_{vl})\Big) \quad (3)$$

Where the first term of Q_m describes the fluid flux entering from the blood vessels and the second term the flux exiting through the lymphatic system. L_p is the blood vessels' hydraulic conductivity, and p_v is the vascular pressure. L_{pl}, S_{vl} and p_{vl} are the corresponding parameters for the lymphatic vessels [23].

Intratumorally injected conjugated-cytokines

$$\frac{\partial Ic_f}{\partial t} + \boldsymbol{\nabla}\cdot\Big(-D_{Ic_f}\boldsymbol{\nabla} Ic_f + \mathbf{v}^f Ic_f\Big) = -\frac{k_{on} c_e Ic_f}{\Phi} + k_{off} Ic_b + Q_{Ic} \quad (4)$$

$$\frac{dIc_b}{\partial t} = \frac{k_{on} c_e Ic_f}{\Phi} - k_{off} Ic_b \quad (5)$$

The free conjugated-cytokines that travel in the tumor interstitial space, Ic_f, can be transferred due to convection and diffusion, where D_{Ic_f} is the diffusion coefficient of the drug in the interstitial space and $\mathbf{v^f}$ is the fluid velocity. Moreover, the free conjugated-cytokines are transferred across the tumor blood vessel and lymphatic vessel wall (Q_{Ic}). The remaining terms describe the binding and unbinding of the conjugated-cytokines: c_e is the concentration of collagen, k_{on}, k_{off} are the binding and unbinding rate constants, respectively and Φ is the volume fraction [14].

Conjugated-cytokines in the blood compartment

The injected conjugated-cytokines concentration in the blood is described as,

$$\frac{Ic_{blood}}{dt} = \frac{\iiint_V -Q_{Ic} dV_{dom}}{V_{blood}} - \delta_{clear} Ic_{blood} \tag{6}$$

Where $\iiint_V -Q_{Ic} dV_{dom}$ is the total rate of mass of the agonist that can be transported from the tumor and host tissue to the blood, V_{blood} is the volume of blood and δ_{clear} the rate of clearance from blood.

Transport from tumor and host tissue to blood

Transport across the vessel and lymphatic vessel wall based on Starling's approximation:

$$Q_{Ic} = P_{er} S_v (Ic_{blood} - Ic_f) + L_p S_v (p_v - p_i)(1 - \sigma_f) Ic_{blood} - \delta_{Ic_f} Ic_f \tag{7}$$

where P_{er} is the vascular permeability of the conjugated-cytokines, σ_f the reflection coefficient and δ_{Ic_f} the rate constant that describes the rate in which the conjugated-cytokines exit through the lymphatic vessels.The parameters L_p, P_{er} and σ_f are expressed as a function of the vessel wall pores and the size of the conjugated-cytokines [7,17]:

$$L_p = \frac{\gamma r_0^2}{8 \mu L_{vw}} \tag{8}$$

$$P_{er} = \frac{\gamma H D_0}{L_{vw}} \tag{9}$$

$$\sigma_f = 1 - W \tag{10}$$

where γ is the fraction of the vessel wall surface area occupied by pores, r_0 the pore radius, μ the viscosity and L_{vw} the thickness of the vessel wall. H and W describe the steric and hydrodynamic interactions of the conjugated-cytokines with the pores of the vessel wall that hinder diffusive and convective transport respectively and D_0 is the diffusion coefficient of a particle in free solution given by the Stokes-Einstein equation. By ignoring electrostatic interactions H and W become [7]:

$$H = \frac{6 \pi F}{K_t} \tag{11}$$

$$W = \frac{F(2 - F) K_s}{2 K_t} \tag{12}$$

where F is the partition coefficient expressed as:

$$F = (1 - \lambda)^2 \tag{13}$$

where λ is the ratio of the conjugated-cytokines size to the vessel wall pore size and K_t and K_s are expressed as [7]:

$$\begin{pmatrix} K_t \\ K_s \end{pmatrix} = \frac{9}{4} \pi^2 \sqrt{2} (1 - \lambda)^{-\frac{5}{2}} \left[1 + \sum_{n=1}^{2} \begin{pmatrix} a_n \\ b_n \end{pmatrix} (1 - \lambda)^n \right] + \sum_{n=0}^{4} \begin{pmatrix} a_{n+3} \\ b_{n+3} \end{pmatrix} \lambda^n \tag{14}$$

Molecular radius of agonist

The radius of the injected agonist is expressed as [22]:

$$\log_{10} R = -0.31 + 0.43 \log_{10} M_w \tag{15}$$

where M_w is the molecular weight of agonist.

Boundary conditions

Needle outlet boundary conditions

$$-\mathbf{n} \cdot \Big(- D_{Ic_f} \boldsymbol{\nabla} Ic_f + \mathbf{v}^f Ic_f \Big) = Ic_{f_{In}} \cdot V_{in} \tag{16}$$

$$\mathbf{v^f} = -V_{in}\mathbf{n} \tag{17}$$

Where V_{in} is the inflow velocity, $Ic_{f_{In}}$ is the initial concentration of injected cytokines.

Needle periphery boundary conditions

$$-\mathbf{n} \cdot \Big(- D_{Ic_f} \boldsymbol{\nabla} Ic_f + \mathbf{v}^f Ic_f \Big) = 0 \tag{18}$$

$$\mathbf{v^f} = 0 \tag{19}$$

External surface boundary conditions

$$Ic_f = 0 \tag{20}$$

$$p_i = 0 \tag{21}$$

Modeling of immune response and tumor growth

Implementation of model equations

The model equations are solved using COMSOL Multiphysics (COMSOL, Inc. Burlington, MA, USA). At the tumor center, the initial cancer cell density was assumed to have its peak value and then it decreases alongside the straight line due to a step function. The domain above a threshold cancer cell density is assumed as the tumor region and below that, the host tissue.
The parameters change from their abnormal-tumor value to their normal value as a function of the cancer cell density. The values change at the threshold cancer cell density using a step function. The step functions are used to certify continuity of the model variables.
Equations were solved at a 1D geometry with spherical symmetry. At the left side of the 1D interval is the tumor center. As we move away from the tumor center we move towards the host tissue. Furthermore, previously used equations 6-15 are also applied on the immune response and tumor growth model.

Kinematics of tumor growth

The growth stretch ratio is calculated as [2]:

$$3\frac{1}{\lambda_g}\frac{d\lambda_g}{dt} = \frac{R_{\overline{T}}}{\overline{T_0}} \tag{22}$$

Where λ_g is the growth stretch ratio $R_{\overline{T}}$ the rate of change of the concentration of cancer cells and $\overline{T_0}$ the initial concentration of cancer cells.
The tumor growth is implemented using deformation of the spatial frame mesh relative to the material frame mesh with a prescribed mesh displacement of

$$\mathbf{d_x} = \mathbf{x} - \mathbf{X} = \lambda_g\mathbf{X} - \mathbf{X} \tag{23}$$

Where $\mathbf{x}$ the spatial frame coordinates and $\mathbf{X}$ the material frame coordinates.
The solid velocity is calculated as:

$$\mathbf{v^s} = \frac{d\mathbf{x}}{dt} \tag{24}$$

Where t is the time.

Interstitial pressure-fluid velocity

Normal and tumor tissues have properties similar to those of a porous medium. According to Darcy's law and the mesh movement due to solid velocity, the interstitial fluid velocity is given by:

$$\mathbf{v^f} = -k_{th}\boldsymbol{\nabla} p_i + \mathbf{v^s} \tag{25}$$

where k_{th} is the hydraulic conductivity of the interstitial space [24]. The mass balance gives [27], [4],

$$\boldsymbol{\nabla}\cdot\mathbf{v^f} = L_pS_v(p_v - p_i) - L_{pl}S_{vl}(p_i - p_{vl}) \tag{26}$$

The first term of the right-hand side of the equation describes the fluid flux entering from the blood vessels and the second term the flux exiting through the lymphatic system. L_p is the blood vessels' hydraulic conductivity, and p_v is the vascular pressure. L_{pl}, S_{vl} and p_{vl} are the corresponding parameters for the lymphatic vessels [23].

Intratumorally injected conjugated-cytokines

$$\frac{\partial Ic_f}{\partial t} + \boldsymbol{\nabla} \cdot \Big(- D_{Ic_f} \boldsymbol{\nabla} Ic_f + \mathbf{v}^f Ic_f \Big) = -\frac{k_{on} c_e Ic_f}{\Phi} + k_{off} Ic_b + Q_{Ic} \tag{27}$$

$$\frac{\partial Ic_b}{\partial t} + \boldsymbol{\nabla} \cdot \Big(\mathbf{v}^{\mathbf{s}} Ic_b \Big) = \frac{k_{on} c_e Ic_f}{\Phi} - k_{off} Ic_b \tag{28}$$

The free conjugated-cytokines that travel in the tumor interstitial space, Ic_f, can be transferred due to convection and diffusion, where D_{Ic_f} is the diffusion coefficient of the conjugated-cytokines in the interstitial space and $\mathbf{v^f}$ is the fluid velocity. Moreover, the free conjugated-cytokines are transferred across the tumor blood vessel and lymphatic vessel wall (Q_{Ic}). The remaining terms describe the binding and unbinding of the conjugated-cytokines: c_e is the concentration of collagen, k_{on}, k_{off} are the binding and unbinding rate constants, respectively and Φ is the volume fraction [14].

Pro-inflammatory cytokines from immune cells

The pro inflammatory cytokines produced by the immune cells can be transported by convection and diffusion:

$$\frac{\partial c_c}{\partial t} + \boldsymbol{\nabla} \cdot \Big(- D_{c_c} \boldsymbol{\nabla} c_c + \mathbf{v}^f c_c \Big) = k_{In} In + k_{T^E} T^E + k_{Th^E} Th^E + k_{APC} APC - \delta_{c_c} c_c \tag{29}$$

Where the right-hand side terms describe the production of pro inflammatory cytokines by innate immune cells, effector CD8+ and CD4+ Tcells and antigen presenting cells. The last term describes the degradation of cytokines.

Total pro-inflammatory cytokines

The total pro-inflammatory cytokines are a combination of the injected cytokines, the injected cytokines bound to collagen and the pro-inflammatory cytokines produced by the immune cells.

$$c = M_w Ic_f + M_w Ic_b + c_c \tag{30}$$

The cytokines related to the injected agonist are solved in $\frac{mol}{m^3}$ and the cytokines by the immune cells in $\frac{kg}{m^3}$ thus the molecular wight M_w converts the units to enable the summation.

Trafficking of immune cells

Normalization of the tumor micro-environment increases trafficking of immune cells when combined with immunotherapy. When the vascular density doubles from $50cm^{-1}$ to $100cm^{-1}$ the source of immune cells increases by 1.367. By assuming that the function is 1 for the host tissue value of ($70cm^{-1}$). The trafficking functions reads pro-inflammatory cytokines produced by the immune cells [18].

$$T_f = 0.0273Sv - 0.9138 \tag{31}$$

where S_v represents the functional vascular density measured in cm^{-1}.

Immature antigen presenting cells

The immature antigen presenting cells are expressed as:

$$\frac{\partial IAPC}{\partial t}+\nabla\cdot\Big(-D_{IAPC}\nabla IAPC+\mathbf{v}^s IAPC\Big)=\lambda_{IAPC}T_f\left(\frac{c}{K_{cAPC}+c}\right)-\delta_{IAPC}IAPC \\ -\chi_{APC}\left(\frac{c}{K_{cAPC}+c}\right)(n_{In}IAPC\overline{T}+n_{A_g}IAPCA_g) \quad (32)$$

Where the first right-hand term describes the source of immature antigen presenting cells, the second term the degradation and the last term the reduction due to activation. The activation to antigen presenting cells depends on the pro-inflammatory cytokines and the interaction of immature antigen presenting cells with the tumor cells and antigen.

Antigen presenting cells

The antigen presenting cells are expressed as:

$$\frac{\partial APC}{\partial t}+\nabla\cdot\Big(-D_{APC}\nabla APC+\mathbf{v}^s APC\Big)= \\ \chi_{APC}\left(\frac{c}{K_{cAPC}+c}\right)(n_{In}IAPC\overline{T}+n_{A_g}IAPCA_g)-\delta_{APC}APC \quad (33)$$

Where the first right-hand term describes the increase of antigen presenting cells due to the activation from immature antigen presenting cells and the last term is a degradation term.

Effector CD4+ T cells

The effector CD4+ T cells are expressed as:

$$\frac{\partial Th^E}{\partial t}+\nabla\cdot\Big(-D_{Th^E}\nabla Th^E+\mathbf{v}^s Th^E\Big)=m_{APC}T_f APC-\delta_{Th^E}Th^E \quad (34)$$

Where the first term describes the source of effector CD4+ T cells which is assumed to be analog to the concentration of antigen presenting cells responsible for the activation of CD4+ T cells in the lymph nodes. The last term is the degradation of CD4+ T cells.

Effector CD8+ T cells

The effector CD8+ T cells are expressed as:

$$\frac{\partial T^E}{\partial t}+\nabla\cdot\Big(-D_{T^E}\nabla T^E+\mathbf{v}^s T^E\Big)=m_{APC}T_f APC-\delta_{T^E}T^E \quad (35)$$

Where the first term describes the source of effector CD8+ T cells which is assumed to be analog to the concentration of antigen presenting cells responsible for the activation of CD8+ T cells in the lymph nodes. The last term is the degradation of CD8+ T cells.

Innate cells

The innate immune cells that induce cytolysis are expressed as:

$$\frac{\partial In}{\partial t}+\nabla\cdot\Big(-D_{In}\nabla In+\mathbf{v}^s In\Big)=\lambda_{In}T_f\left(\frac{c}{K_{cAPC}+c}\right)-\delta_{In}In \quad (36)$$

Where the first term describes production of innate cells which depends on the concentration of pro-inflammatory cytokines.

Cancer cells

The concentration of cancer cells is expressed as:

$$\frac{\partial \overline{T}}{\partial t} + \boldsymbol{\nabla} \cdot \Big(- D_{\overline{T}} \boldsymbol{\nabla} \overline{T} + \mathbf{v}^s \overline{T} \Big) = R_{\overline{T}} = k_1 \left(\frac{c_{ox}}{K_2 + c_{ox}} \right) \overline{T} - (n_{In} In + n_{In} IAPC + n_{ad} T^E) \overline{T} \quad (37)$$

Where the first right-hand side term describes the proliferation of cancer cells due to oxygen and the last term describes the killing of cancer cells by innate cells, immature antigen presenting cells and effector CD8+ T cells.

Antigen

The concentration of cancer cells is expressed as:

$$\frac{\partial A_g}{\partial t} + \boldsymbol{\nabla} \cdot \Big(- D_{A_g} \boldsymbol{\nabla} A_g + \mathbf{v}^f A_g \Big) = (n_{In} In + n_{ad} T^E) \overline{T} - n_{A_g} IAPC A_g \quad (38)$$

Where the first right-hand side term describes the production of antigen induced by the cytolytic effect of innate cells and effector CD8+ T cells. The last term describes the antigen uptake by the immature antigen presenting cells.

Oxygen transport

The rate of change of oxygen concentration in the tissue was modeled with a convection diffusion equation that includes a source and a sink term [20], [11]. The source term is due to oxygen supply from the blood vessels and the sink term describes oxygen consumption by cancer cells:

$$\frac{\partial c_{ox}}{\partial t} + \boldsymbol{\nabla} \cdot \Big(- D_{c_{ox}} \boldsymbol{\nabla} c_{ox} + \mathbf{v}^f c_{ox} \Big) = - \frac{A_{ox} c_{ox}}{c_{ox} + k_{ox}} \frac{\overline{T}}{\overline{T_0}} + P_{erox} S_v (c_{iox} - c_{ox}) \quad (39)$$

where S_v is the vascular density, D_{ox} the oxygen diffusion coefficient, A_{ox} and k_{ox} are oxygen uptake parameters, c_{iox} is the oxygen concentration in the vessels, $\mathbf{v}^f$ is the fluid velocity and P_{erox} is the vascular permeability of oxygen defined as the oxygen diffusion coefficient divided by the length of the vessel wall.

Immune cell death rate

According to experimental data [3], a 40 times decrease in oxygen concentration (from 20% to 0.5%) doubled the apoptotic rate of immune cells. Thus the degradation rates are expressed as:

$$\delta_{IAPC} = \delta_{IAPC_0} + 1.025 \cdot (1 - \frac{c_{ox}}{c_{iox}}) \cdot \delta_{IAPC_0} \quad (40)$$

$$\delta_{APC} = \delta_{APC_0} + 1.025 \cdot (1 - \frac{c_{ox}}{c_{iox}}) \cdot \delta_{APC_0} \quad (41)$$

$$\delta_{Th^E} = \delta_{Th^E_0} + 1.025 \cdot (1 - \frac{c_{ox}}{c_{iox}}) \cdot \delta_{Th^E_0} \quad (42)$$

$$\delta_{T^E} = \delta_{T^E_0} + 1.025 \cdot (1 - \frac{c_{ox}}{c_{iox}}) \cdot \delta_{T^E_0} \quad (43)$$

$$\delta_{In} = \delta_{In_0} + 1.025 \cdot (1 - \frac{c_{ox}}{c_{iox}}) \cdot \delta_{In_0} \quad (44)$$

Boundary conditions

Tumor center boundary conditions

Due to the symmetry at the tumor center no flux boundary conditions were used thus no diffusive or convective transport. No flux boundary conditions were applied to all partial differential equations.

$$-\mathbf{n}\cdot\Big(-D_{c_{ox}}\boldsymbol{\nabla}c_{ox}+\mathbf{v}^f c_{ox}\Big)=0 \tag{45}$$

$$-\mathbf{n}\cdot\Big(-k_{th}\boldsymbol{\nabla}p_i+\mathbf{v}^s\Big)=0 \tag{46}$$

$$-\mathbf{n}\cdot\Big(-D_{In}\boldsymbol{\nabla}In+\mathbf{v}^s In\Big)=0 \tag{47}$$

$$-\mathbf{n}\cdot\Big(-D_{IAPC}\boldsymbol{\nabla}IAPC+\mathbf{v}^s IAPC\Big)=0 \tag{48}$$

$$-\mathbf{n}\cdot\Big(-D_{c_c}\boldsymbol{\nabla}c_c+\mathbf{v}^s c_c\Big)=0 \tag{49}$$

$$-\mathbf{n}\cdot\Big(-D_{T^E}\boldsymbol{\nabla}T^E+\mathbf{v}^s T^E\Big)=0 \tag{50}$$

$$-\mathbf{n}\cdot\Big(-D_{Th^E}\boldsymbol{\nabla}Th^E+\mathbf{v}^s Th^E\Big)=0 \tag{51}$$

$$-\mathbf{n}\cdot\Big(-D_{APC}\boldsymbol{\nabla}APC+\mathbf{v}^s APC\Big)=0 \tag{52}$$

$$-\mathbf{n}\cdot\Big(-D_{\overline{T}}\boldsymbol{\nabla}\overline{T}+\mathbf{v}^s \overline{T}\Big)=0 \tag{53}$$

$$-\mathbf{n}\cdot\Big(-D_{A_g}\boldsymbol{\nabla}A_g+\mathbf{v}^s A_g\Big)=0 \tag{54}$$

$$-\mathbf{n}\cdot\Big(-D_{Ic_f}\boldsymbol{\nabla}Ic_f+\mathbf{v}^s Ic_f\Big)=0 \tag{55}$$

$$-\mathbf{n}\cdot\Big(\mathbf{v}^s Ic_b\Big)=0 \tag{56}$$

External surface boundary conditions

For the external surface boundary condition. Oxygen was assumed to have a constant value of the normal tissue.

$$c_{ox}=c_{iox} \tag{57}$$

No flux boundary condition was applied for the solution of the fluid pressure, according to the Darcy law and the domain deformation by the solid velocity.

$$-\mathbf{n}\cdot\Big(-k_{th}\boldsymbol{\nabla}p_i+\mathbf{v}^s\Big)=0 \tag{58}$$

No flux boundary conditions were applied for the innate immune cells and the cytokines produced by the immune cells.

$$-\mathbf{n}\cdot\Big(-D_{In}\boldsymbol{\nabla}In+\mathbf{v}^s In\Big)=0 \tag{59}$$

$$-\mathbf{n}\cdot\Big(-D_{IAPC}\boldsymbol{\nabla}IAPC+\mathbf{v}^s IAPC\Big)=0 \tag{60}$$

$$-\mathbf{n}\cdot\Big(-D_{c_c}\boldsymbol{\nabla}c_c+\mathbf{v}^s c_c\Big)=0 \tag{61}$$

The external surface is away from the tumor region and it is not reached by the cancer cells. Thus the cancer cells density was assumed 0 as well as all the effector cells and the antigen that depend on the existence of tumor cells.

$$T^E = 0 \tag{62}$$

$$Th^E = 0 \tag{63}$$

$$APC = 0 \tag{64}$$

$$\overline{T} = 0 \tag{65}$$

$$A_g = 0 \tag{66}$$

The external surface is away from the tumor region and thus it is not reached by the injected cytokines. Thus the injected cytokines and the cytokines that bound to target were assumed 0.

$$Ic_f = 0 \tag{67}$$

$$Ic_b = 0 \tag{68}$$

Supplementary figures

Intratumoral injection model computational domain

Figure A. Computational domain with axial symmetry. The domain includes the tumor region and the host tissue. The needle reaches the center of a spherical tumor.

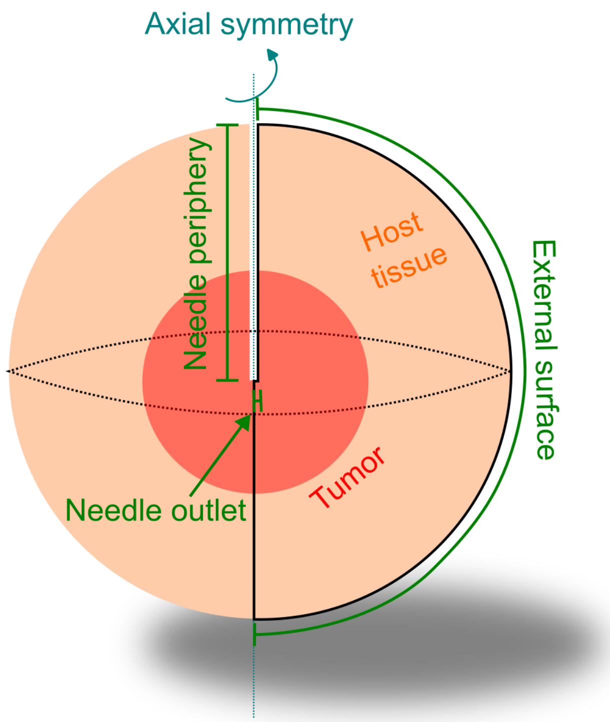

Immune response and tumor growth model computational domain

Figure B. Computational domain with spherical symmetry. The interval includes the tumor region and the host tissue. The 1D tumor interval forms a 3D spherical tumor. The tumor grows as a sphere and deforms the host tissue.

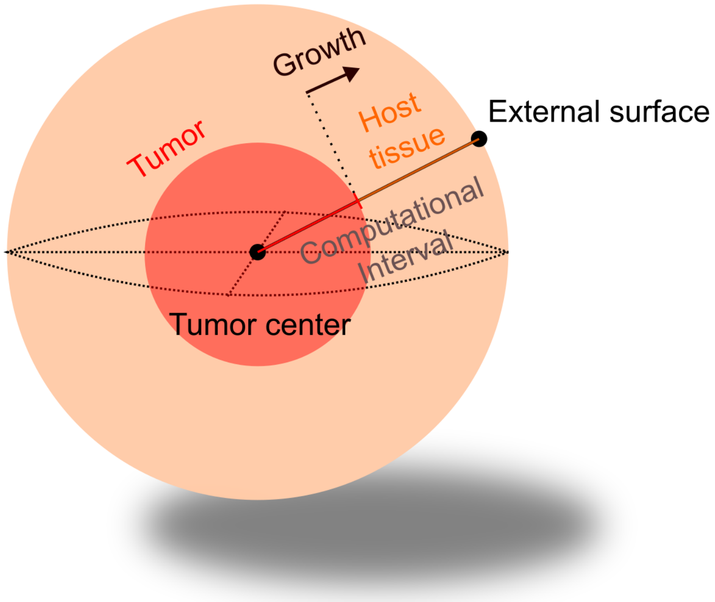

Fitting of the model to experimental data

For each fitting we calculated the sum of the squared difference of the values between the measured V_{exp} and simulated V_{calc} tumor volumes divided by the number of experimental values N,

$$Er = \frac{\sum_i [V_{exp,i} - V_{calc,i}]^2}{N} \tag{69}$$

We repeated this calculation for all fittings in order to calculate Er_{tot}.

$$Er_{tot} = Er_{exp1,control} + Er_{exp2,control} + Er_{exp1,drug} + Er_{exp2,drug} \tag{70}$$

When Er_{tot} becomes zero, the simulated volume curves match exactly with the experimental points. Thus, we minimize this value using the COMSOL with MATLAB interface (LiveLink for MATLAB) in order for Matlab to find the optimum parameters that can be used in the COMSOL model.

Supplementary tables

Model characteristics

Table A. Mathematical model characteristics compared to other models

Model Characteristic	Momin et al [16]	This study
Conjugated-cytokines size - molecular weight	✓	✓
Conjugated-cytokines affinity	✓	✓
Conjugated-cytokines binding and unbinding to target	✓	✓
Conjugated-cytokines intravasation and extravasation	✓	✓
Conjugated-cytokines cleared from blood	✓	✓
Temporal changes of conjugated-cytokines concentration	✓	✓
The injected protein binds on IL-2 receptor (IL-2R)	✓	✗
Modeling the injection of conjugated-cytokines from the needle to the tissue	✗	✓
Spatial changes of cytokines concentration	✗	✓
Convective transport of cytokines	✗	✓
Transport to the surrounding host tissue	✗	✓
Tumor pathophysiology (tumor vasculature, interstitial fluid pressure distribution, lymphatic system, hydraulic conductivity)	✗	✓
Effect of the treatment to tumor growth	✗	✓
Immune cells and cancer cells interaction	✗	✓

Model variables

Table B. Table of model variables

Description	Variable
Interstitial fluid pressure	p_i
Fluid velocity	$\mathbf{v^f}$
Solid velocity	$\mathbf{v^b}$
Free conjugated-cytokines	Ic_f
Conjugated-cytokines bound	Ic_b
Conjugated-cytokines in the blood	Ic_{blood}
Growth stretch ratio	λ_g
Pro-inflammatory cytokines produced from immune cells	c_c
Total pro-inflammatory cytokines	c
Antigen presenting cells	APC
Effector CD4+ T cells	Th^E
Effector CD8+ T cells	T^E
Innate cells	In
Cancer cells	$\overline{T}$
Antigen	A_g
Oxygen	c_{ox}

Model parameters

Table C. Table of model parameters

Description	Parameter	Value	Reference
Hydraulic conductivity (tumor)	k_{thT}	$4.13 \cdot 10^{-8}\ cm^2 \cdot mmHg^{-1} \cdot s^{-1}$	[4]
Hydraulic conductivity (host)	k_{thH}	$8.53 \cdot 10^{-9}\ cm^2 \cdot mmHg^{-1} \cdot s^{-1}$	[4]
Oxygen concentration in the vessels	c_{iox}	$0.2\ mol \cdot m^{-3}$	[9,11]
Oxygen diffusion coefficient	D_{ox}	$1.55 \cdot 10^{-4}\ m^2 \cdot day^{-1}$	[11]
Oxygen uptake parameter A	A_{ox}	$2200\ mol \cdot m^{-3} \cdot day^{-1}$	[5,11]
Oxygen uptake parameter k	k_{ox}	$0.00464\ mol \cdot m^{-3}$	[5,11]
Growth rate parameter	k_2	$0.0083\ mol \cdot m^{-3}$	[5]
Vascular pressure	p_v	$15.6\ mmHg$	[4]
Lymphatic pressure	p_{vl}	0	[23]
Vascular density (host)	S_{vH}	$70\ cm^{-1}$	[26]
Vascular density (tumor)	S_{vT}	$50\ cm^{-1}$	[26]
Permeability of lymphatics	$L_{pl}S_{vl}$	$0.05\ mmHg^{-1} \cdot s^{-1}$	[4]
Vessel wall thickness	L_{vw}	$5 \cdot 10^{-6}\ m$	[23]
Water viscosity at 310K	vis	$7 \cdot 10^{-4}\ Pa \cdot s$	[23]
Fraction of vessel wall surface area occupied by pores (tumor)	γ_T	10^{-3}	[6]
Fraction of vessel wall surface area occupied by pores (host)	γ_H	10^{-4}	[6]
Vessel wall pore radius (host)	r_{0H}	$3.5\ nm$	[10]
Vessel wall pore radius (tumor)	r_{0T}	$100\ nm$	[10]
Production of proinflammatory cytokines by innate immune cells	k_{In}	$3 \cdot 10^{-8}\ day^{-1}$	[8]
Production of proinflammatory cytokines by effector CD8+ Tcells	k_{T^E}	$3 \cdot 10^{-8}\ day^{-1}$	[8]

Production of proinflammatory cytokines by effector CD4+ Tcells	k_{Th^E}	$3 \cdot 10^{-8}\ day^{-1}$	[8]
Production of proinflammatory cytokines by antigen presenting cells	k_{APC}	$3{\cdot}10^{-8}\ day^{-1}$	[8]
Initial degradation of antigen presenting cells	δ_{APC_0}	$0.1\ day^{-1}$	[8]
Initial degradation of immature antigen presenting cells	δ_{IAPC_0}	$0.1\ day^{-1}$	[8]
Initial degradation of effector CD4+ Tcells	$\delta_{Th_0^E}$	$0.197\ day^{-1}$	[8]
Initial degradation of effector CD8+ Tcells	$\delta_{T_0^E}$	$0.18\ day^{-1}$	[8]
Initial degradation of Innate immune cells	δ_{In_0}	$0.18\ day^{-1}$	[8]
Degradation of cytokines produced by immune cells	δ_{c_c}	$1.38\ day^{-1}$	[8]
The rate in which the drug exits through the lymphatic vessels (host)	$\delta_{Ic_{flH}}$	$1.38\ day^{-1}$	[8]
The rate in which the drug exits through the lymphatic vessels (tumor)	$\delta_{Ic_{flT}}$	0	[17]
Production of APCs	χ_{APC}	$0.5\ cm^3 \cdot g^{-1}$	This study
Growth rate parameter (Agarwal et. al. [1])	k_{1A}	$0.19409\ day^{-1}$	Optimization
Growth rate parameter (Momin et. al. [15])	k_{1M}	$0.3446\ day^{-1}$	Optimization
Source of effector CD4+ and source of effector CD8+ (Momin et. al. [15])	m_{APCM}	$2.36 \cdot 10^{-6}\ s^{-1}$	Optimization
Production of NK (Momin et. al. [15])	λ_{InM}	$0.025\ g \cdot cm^{-3} \cdot day^{-1}$	Optimization
Production of IAPC (Momin et. al. [15])	λ_{IAPCM}	$0.025\ g \cdot cm^{-3} \cdot day^{-1}$	Optimization

Half saturation concentration Innate cells (Momin et. al. [15])	K_{cInM}	$9.1346 \cdot 10^{-4}\ g \cdot cm^{-3}$	Optimization
Half saturation antigen presenting cells (Momin et. al. [15])	K_{cAPCM}	$9.1346 \cdot 10^{-4}\ g \cdot cm^{-3}$	Optimization
Killing rate constants of tumor cells by innate immune cells (Momin et. al. [15])	n_{InM}	$54.1857\ cm^3 \cdot g^{-1} \cdot day^{-1}$	Optimization
killing rate constants of tumor cells by adaptive immune cells (Momin et. al. [15])	n_{adM}	$108.3713\ cm^3 \cdot g^{-1} \cdot day^{-1}$	Optimization
Antigen uptake rate (Momin et. al. [15])	n_{A_gM}	$108.3713\ cm^3 \cdot g^{-1} \cdot day^{-1}$	Optimization
Source of effector CD4+ and source of effector CD8+ (Agarwal et. al. [1])	m_{APCA}	$2.1395 \cdot 10^{-6}\ s^{-1}$	Optimization
Production of NK (Agarwal et. al. [1])	λ_{InA}	$0.0253\ g \cdot cm^{-3} \cdot day^{-1}$	Optimization
Production of IAPC (Agarwal et. al. [1])	λ_{IAPCA}	$0.0253\ g \cdot cm^{-3} \cdot day^{-1}$	Optimization
Half saturation concentration Innate cells (Agarwal et. al. [1])	K_{cInA}	$7.0143 \cdot 10^{-4}\ g \cdot cm^{-3}$	Optimization
Half saturation antigen presenting cells (Agarwal et. al. [1])	K_{cAPCA}	$7.0143 \cdot 10^{-4}\ g \cdot cm^{-3}$	Optimization
Killing rate constants of tumor cells by innate immune cells (Agarwal et. al. [1])	n_{InA}	$44.0016\ cm^3 \cdot g^{-1} \cdot day^{-1}$	Optimization
Killing rate constants of tumor cells by adaptive immune cells (Agarwal et. al. [1])	n_{adA}	$88.0032\ cm^3 \cdot g^{-1} \cdot day^{-1}$	Optimization
Antigen uptake rate (Agarwal et. al. [1])	n_{A_gA}	$88.0032\ cm^3 \cdot g^{-1} \cdot day^{-1}$	Optimization
Diffusion coefficient IAPC	D_{IAPC}	$4.4150 \cdot 10^{-4}\ cm^2 \cdot day^{-1}$	[25]
Diffusion coefficient APC	D_{APC}	$4.4150 \cdot 10^{-4}\ cm^2 \cdot day^{-1}$	[25]

Diffusion coefficient CD4	D_{Th^E}	$4.4150 \cdot 10^{-4}\ cm^2 \cdot day^{-1}$	[25]
Diffusion coefficient CD8	D_{T^E}	$4.4150 \cdot 10^{-4}\ cm^2 \cdot day^{-1}$	[25]
Diffusion coefficient Innate immune cells	D_{In}	$4.4150 \cdot 10^{-4}\ cm^2 \cdot day^{-1}$	[25]
Diffusion cancer cells	$D_{\overline{T}}$	$4.4150 \cdot 10^{-4}\ cm^2 \cdot day^{-1}$	[25]
Diffusion coefficient pro-inflamatory cytokines by immune cells	D_{c_c}	$6.0472 \cdot 10^{-2}\ cm^2 \cdot day^{-1}$	[12]
Diffusion coefficient Antigen	D_{A_g}	$6.0472 \cdot 10^{-2}\ cm^2 \cdot day^{-1}$	[12]
Volume of blood	V_{blood}	$2\ mL$	[16]
Volume fraction of tumor accessible to drug	Φ	0.3	[14, 21]
Initial concentration of pro inflammatory cytokines produced by immune cells	c_{cin}	$3 \cdot 10^{-11}\ g \cdot cm^{-3}$	[8]
Initial concentration of Innate immune cells	In_{In}	$9 \cdot 10^{-4}\ g \cdot cm^{-3}$	[8]
Initial concentration of Immature antigen presenting cells	$IAPC_{In}$	$5 \cdot 10^{-5}\ g \cdot cm^{-3}$	[8]
Initial concentration of tumor cells	$\overline{T_0}$	$0.4\ g \cdot cm^{-3}$	[8]
Tumor region threshold concentration	$Thld$	$0.2\ g \cdot cm^{-3}$	This study
Concentration of surface receptors	c_e	$2 \cdot 10^{-4}\ mol \cdot m^{-3}$	[16]
Binding rate constant	k_{on}	$1 \cdot 10^{2}\ m^3 \cdot mol^{-1} \cdot s^{-1}$	[16]
Dissociation rate constant	k_{off}	$1 \cdot 10^{-3}\ s^{-1}$	[16]
Density	ρ	$1000\ kg \cdot m^{-3}$	[28]
Inflow velocity	V_{in}	$0.075215\ m \cdot s^{-1}$	This study
Porosity	ϵ_p	0.3	[13]
Initial concentration of injected cytokines (Momin et. al. [15])	$Ic_{f_{InM}}$	$0.082645\ mol \cdot m^{-3}$	[16]

Initial concentration of injected cytokines (Agarwal et. al. [1])	$Ic_{f_{InA}}$	$0.01\ mol \cdot m^{-3}$	[1]
Molecular weight of agonist (Momin et. al. [15])	M_{wM}	$121kDa$	[15]
Diffusion coefficient injected pro-inflamatory cytokines free (Momin et. al. [15])	D_{Ic_fM}	$0.5 \cdot 10^{-6}\ cm^2 \cdot s^{-1}$	[19]
Rate of clearance of agonist (Momin et. al. [15])	δ_{clearM}	$0.4\ hr^{-1}$	[21]
Initial tumor radius (Momin et. al. [15])	$TumorX_M$	$2.3489\ mm$	[15]
Molecular weight of agonist (Agarwal et. al. [1])	M_{wA}	$65kDa$	[1]
Diffusion coefficient injected pro-inflamatory cytokines free (Agarwal et. al. [1])	D_{Ic_fA}	$0.75 \cdot 10^{-6}\ cm^2 \cdot s^{-1}$	[19]
Rate of clearance of agonist (Agarwal et. al. [1])	δ_{clearA}	$1.5\ hr^{-1}$	[21]
Initial tumor radius (Agarwal et. al. [1])	$TumorX_A$	$2.8338\ mm$	[1]

References

1. Yash Agarwal, Lauren E. Milling, Jason Y.H. Chang, Luciano Santollani, Allison Sheen, Emi A. Lutz, Anthony Tabet, Jordan Stinson, Kaiyuan Ni, Kristen A. Rodrigues, Tyson J. Moyer, Mariane B. Melo, Darrell J. Irvine, and K. Dane Wittrup. Intratumourally injected alum-tethered cytokines elicit potent and safer local and systemic anticancer immunity. *Nature Biomedical Engineering 2022 6:2*, 6:129–143, 1 2022.

2. Stelios Angeli, Kyrre E. Emblem, Paulina Due-Tonnessen, and Triantafyllos Stylianopoulos. Towards patient-specific modeling of brain tumor growth and formation of secondary nodes guided by dti-mri. *NeuroImage: Clinical*, 20:664–673, 1 2018.

3. Ivraym B. Barsoum, Chelsea A. Smallwood, D. Robert Siemens, and Charles H. Graham. A mechanism of hypoxia-mediated escape from adaptive immunity in cancer cells. *Cancer Research*, 74:665–674, 2 2014.

4. Laurence T. Baxter and Rakesh K. Jain. Transport of fluid and macromolecules in tumors. i. role of interstitial pressure and convection. *Microvascular Research*, 37:77–104, 1 1989.

5. Joseph J. Casciari, Stratis V. Sotirchos, and Robert M. Sutherland. Variations in tumor cell growth rates and metabolism with oxygen concentration, glucose concentration, and extracellular ph. *Journal of Cellular Physiology*, 151:386–394, 5 1992.

6. Vikash P. Chauhan, Triantafyllos Stylianopoulos, John D. Martin, Zoran PopoviÄ, Ou Chen, Walid S. Kamoun, Moungi G. Bawendi, Dai Fukumura, and Rakesh K. Jain. Normalization of tumour blood vessels improves the delivery of nanomedicines in a size-dependent manner. *Nature Nanotechnology*, 7:383–388, 2012.

7. W. M. Deen. Hindered transport of large molecules in liquid[U+2010]filled pores. *AIChE Journal*, 33:1409–1425, 9 1987.

8. Avner Friedman and Wenrui Hao. The role of exosomes in pancreatic cancer microenvironment. *Bulletin of Mathematical Biology 2017 80:5*, 80:1111–1133, 4 2017.

9. Patrick C. Hermann, Stephan L. Huber, Tanja Herrler, Alexandra Aicher, Joachim W. Ellwart, Markus Guba, Christiane J. Bruns, and Christopher Heeschen. Distinct populations of cancer stem cells determine tumor growth and metastatic activity in human pancreatic cancer. *Cell Stem Cell*, 1:313–323, 9 2007.

10. Susan K. Hobbs, Wayne L. Monsky, Fan Yuan, W. Gregory Roberts, Linda Griffith, Vladimir P. Torchilin, and Rakesh K. Jain. Regulation of transport pathways in tumor vessels: Role of tumor type and microenvironment. *Proceedings of the National Academy of Sciences*, 95:4607–4612, 4 1998.

11. Yangjin Kim, Magdalena A. Stolarska, and Hans G. Othmer. The role of the microenvironment in tumor growth and invasion. *Progress in Biophysics and Molecular Biology*, 106:353–379, 8 2011.

12. Xiulan Lai and Avner Friedman. Combination therapy of cancer with cancer vaccine and immune checkpoint inhibitors: A mathematical model. *PLOS ONE*, 12:e0178479, 5 2017.

13. Chryso Lambride, Vasileios Vavourakis, and Triantafyllos Stylianopoulos. Convection-enhanced delivery in silico study for brain cancer treatment. *Frontiers in Bioengineering and Biotechnology*, 10:841, 5 2022.

14. Wilson Mok, Triantafyllos Stylianopoulos, Yves Boucher, and Rakesh K. Jain. Mathematical modeling of herpes simplex virus distribution in solid tumors: Implications for cancer gene therapy. *Clinical Cancer Research*, 15:2352–2360, 4 2009.

15. Noor Momin, Naveen K. Mehta, Nitasha R. Bennett, Leyuan Ma, Joseph R. Palmeri, Magnolia M. Chinn, Emi A. Lutz, Byong Kang, Darrell J. Irvine, Stefani Spranger, and K. Dane Wittrup. Anchoring of intratumorally administered cytokines to collagen safely potentiates systemic cancer immunotherapy. *Science Translational Medicine*, 11, 6 2019.

16. Noor Momin, Joseph R. Palmeri, Emi A. Lutz, Noor Jailkhani, Howard Mak, Anthony Tabet, Magnolia M. Chinn, Byong H. Kang, Virginia Spanoudaki, Richard O. Hynes, and K. Dane Wittrup. Maximizing response to intratumoral immunotherapy in mice by tuning local retention. *Nature Communications 2022 13:1*, 13:1–13, 1 2022.

17. Fotios Mpekris, Stelios Angeli, Athanassios P. Pirentis, and Triantafyllos Stylianopoulos. Stress-mediated progression of solid tumors: effect of mechanical stress on tissue oxygenation, cancer cell proliferation, and drug delivery. *Biomechanics and Modeling in Mechanobiology*, 14:1391–1402, 11 2015.

18. Myrofora Panagi, Fotios Mpekris, Chrysovalantis Voutouri, Christina Michael, Anastasia Constantinidou, John D. Martin, and Triantafyllos Stylianopoulos. Abstract 6382: Targeting mast cells restores T cell infiltration and sensitizes sarcomas to PD-L1 inhibition. *Cancer Research*, 82:6382–6382, 06 2022.

19. Alain Pluen, Yves Boucher, Saroja Ramanujan, Trevor D. McKee, Takeshi Gohongi, Emmanuelle Di Tomaso, Edward B. Brown, Yotaro Izumi, Robert B. Campbell, David A. Berk, and Rakesh K. Jain. Role of tumor-host interactions in interstitial diffusion of macromolecules: Cranial vs. subcutaneous tumors. *Proceedings of the National Academy of Sciences of the United States of America*, 98:4628–4633, 4 2001.

20. Tiina Roose, Paolo A. Netti, Lance L. Munn, Yves Boucher, and Rakesh K. Jain. Solid stress generated by spheroid growth estimated using a linear poroelasticity model. *Microvascular Research*, 66:204–212, 11 2003.

21. Michael M. Schmidt and K. Dane Wittrup. A modeling analysis of the effects of molecular size and binding affinity on tumor targeting. *Molecular Cancer Therapeutics*, 8:2861–2871, 10 2009.

22. Detlef M. Smilgies and Ewa Folta-Stogniew. Molecular weight–gyration radius relation of globular proteins: a comparison of light scattering, small-angle x-ray scattering and structure-based data. *urn:issn:1600-5767*, 48:1604–1606, 9 2015.

23. Triantafyllos Stylianopoulos, John D. Martin, Matija Snuderl, Fotios Mpekris, Saloni R. Jain, and Rakesh K. Jain. Coevolution of solid stress and interstitial fluid pressure in tumors during progression: Implications for vascular collapse. *Cancer Research*, 73:3833–3841, 7 2013.

24. Triantafyllos Stylianopoulos, Andrew Yeckel, Jeffrey J. Derby, Xiao Juan Luo, Mark S. Shephard, Edward A. Sander, and Victor H. Barocas. Permeability calculations in three-dimensional isotropic and oriented fiber networks. *Physics of Fluids*, 20:123601, 12 2008.

25. Chrysovalantis Voutouri, Nathaniel D. Kirkpatrick, Euiheon Chung, Fotios Mpekris, James W. Baish, Lance L. Munn, Dai Fukumura, Triantafyllos Stylianopoulos, and Rakesh K. Jain. Experimental and computational analyses reveal dynamics of tumor vessel cooption and optimal treatment strategies. *Proceedings of the National Academy of Sciences of the United States of America*, 116:2662–2671, 2 2019.

26. Chrysovalantis Voutouri, Fotios Mpekris, Panagiotis Papageorgis, Andreani D. Odysseos, and Triantafyllos Stylianopoulos. Role of constitutive behavior and tumor-host mechanical interactions in the state of stress and growth of solid tumors. *PLoS ONE*, 9:e104717, 8 2014.

27. Mostafa Zakariapour, Mohammad Hossein Hamedi, and Nasser Fatouraee. Características do fluxo de fluido intersticial junto com o fluxo sanguíneo no interior de um tumor cilíndrico: Uma simulação numérica. *Acta Scientiarum - Technology*, 40, 2018.

28. Wenbo Zhan and Chi Hwa Wang. Convection enhanced delivery of liposome encapsulated doxorubicin for brain tumour therapy. *Journal of Controlled Release*, 285:212–229, 9 2018.
